# Shrinking Alpine chamois: higher spring temperatures over the last 27 years in Switzerland are linked to a 3 kg reduction in body mass of yearlings

**DOI:** 10.1098/rsos.231295

**Published:** 2024-03-13

**Authors:** Giulia Masoero, Kristina Georgieva Gencheva, Noémie Ioset, Louis-Félix Bersier, Federico Tettamanti, Pierre Bize

**Affiliations:** ^1^ Department of Biology, University of Ottawa, Ottawa, Canada; ^2^ Swiss Ornithological Institute, Seerose 1, Sempach 6204, Switzerland; ^3^ School of Biological Sciences, University of Aberdeen, Aberdeen, UK; ^4^ Department of Biology, University of Fribourg, Chemin du Musée 10, Fribourg, Switzerland; ^5^ Studio alpino Tettamanti, La Campagna d Zora 15, Lodano 6678, Switzerland; ^6^ Ufficio della Caccia e della Pesca del Cantone Ticino, Bellinzona, Switzerland

**Keywords:** climate change, *climwin*, ungulates, life stages, temperature, elevation

## Abstract

Although climate change is considered to be partly responsible for the size change observed in numerous species, the relevance of this hypothesis for ungulates remains debated. We used body mass measurements of 5635 yearlings (i.e. 1.5 years old) of Alpine chamois (*Rupicapra rupicapra*) harvested in September in the Swiss Alps (Ticino canton) from 1992 to 2018. In our study area, during this period, yearlings shrank by *ca* 3 kg while temperatures between May and July rose by 1.7°C. We identified that warmer temperatures during birth and the early suckling period (9 May to 2 July in the year of birth) had the strongest impact on yearling mass. Further analyses of year-detrended mass and temperature data indicate that this result was not simply due to changes in both variables over years, but that increases in temperature during this particularly sensitive time window for development and growth are responsible for the decrease in body mass of yearling chamois. Altogether, our results suggest that rising temperatures in the Alpine regions could significantly affect the ecology and evolution of this wild ungulate.

## 1. Introduction

As global changes induced by human activities accelerate, many species are undergoing phenotypic changes to adapt to their new environment [1], often measured by changes in distribution, abundance and phenology. An increasingly reported response to climate warming is the change in animal body size and shape [2–4], as morphology affects thermoregulation [5]. Indeed, a shrinking in body size leads to a larger surface-area-to-volume ratio, which, in turn, allows animals to dissipate heat more efficiently in warmer environments (Bergmann’s rule; e.g. [6]). Global warming is also likely to impact body size due to changes in food availability and quality [7]. For example, the increase in frequency and length of summer droughts is expected to reduce forage quality and palatability for grazing species [8–10]. In mammals, high ambient temperature, together with the limits to the ability to dissipate heat, has also been previously shown to constrain maternal milk production and offspring growth [5,11], limiting adult size.

The size an individual reaches as an adult has critical consequences for reproductive success and overall fitness [12]. As adult size and mass are primarily determined by early growth conditions and juvenile size (e.g. [13]), it is fundamental to investigate how individuals are affected by climate in early-life stages [14]. Because juveniles usually have low energy reserves and must use a large portion of them for growth [15,16], they are sensitive to external biotic and abiotic factors [17–19]. In mammals, early growth is divided into three phases: gestation, suckling and post-weaning. However, the three phases might not be equally sensitive to climate warming. For example, the suckling phase might be the most sensitive, as offspring growth is the fastest during suckling, and the mother’s milk production is constrained by high ambient temperatures [5,11].

We investigated the effect of weather conditions on the yearling size (i.e. 1.5 years of age) of Alpine chamois (*Rupicapra rupicapra*) using hunting data collected in the southern Swiss Alps from 1992 to 2018. We aimed to describe the body mass decrease in yearlings and identify which temporal period of their growth is most sensitive to temperature conditions. The Alpine chamois is the most abundant ungulate of the European Alps [20], and its morphology and physiology are adapted to high-elevation (cold) environmental conditions [21]. Accordingly, most previous studies on the Alpine chamois have revealed a gradual shrinking in chamois body mass (e.g. [19,22,23], but see [24]). As observed previously by these studies, we expected a temporal decrease in the body mass of yearling chamois and a negative relationship with the temperature during spring–summer in the first two years of life (e.g. [19]). To our knowledge, no study has yet precisely identified which developmental window during early life is most sensitive to climate warming and whether the shrinking in body mass over time is associated with an increase in temperature during this critical developmental window.

## 2. Material and methods

### 2.1. Study system

The Alpine chamois is a medium-sized ungulate with an asymptotic body mass of 22.3 kg in females and 29.6 kg in males [25–27]. In the Alps, chamois give birth in May [28] and suckle their offspring from May to July. These months also correspond to the growth peak of yearlings in their second year of life. Chamois are weaned between three and six months of age [29]. Vegetation in the Alps usually begins growing right after snow-melt in April, peaking in July, thus providing an abundant and protein-rich food source for a relatively brief period of time [30]. Alpine chamois are distributed over a broad altitudinal range (500–3100 m.a.s.l.; e.g. [31,32]) and generally follow resource availability and climate conditions [23,33].

Data on chamois size were extracted from hunting records in the southern Alps of Switzerland (canton Ticino) between 1992 and 2018. The study area covers 2700 km^2^ with an elevation range of 250*–*2700 m.a.s.l. In Ticino, hunting starts at the beginning of September and is completed within 10 days. As required by Swiss law, animals were eviscerated by hunters, and their entrails were left at the kill site. Carcasses were then brought to checkpoints in the Ticino canton within 48 hours, where trained gamekeepers and wildlife wardens sexed and aged the animals before recording their weight with an electronic precision scale to the nearest 0.1 kg (eviscerated body weight). Age is estimated by counting the growth rings of horns [34]. The use of the eviscerated body weight is a good proxy for the total mass (skeletal, muscle and fat store) of the animal, as it excludes the variation due to recently ingested food. Coordinates of the kill locations were provided by hunters at the official checkpoints when weighing their hunt, and elevation was extracted *a posteriori*. Of 34 017 animals harvested during the hunting period (age range: 0.5*–*22.5 years old), 5635 were yearlings (1.5 years old; 2491 females and 3144 males).

Daily mean ambient temperatures (℃) from 1990 until 2018 (all years needed for analysis) were obtained from a Swiss meteorological station in the city of Lugano (273 m.a.s.l.), located within the harvesting area. Other stations at higher elevations had incomplete data but showed a high correlation with Lugano data (all Pearson *r* > 0.8; electronic supplementary material, S1), indicating that Lugano provides a good proxy for temperature in our study area.

### 2.2. Statistical analysis

**Figure 1.**
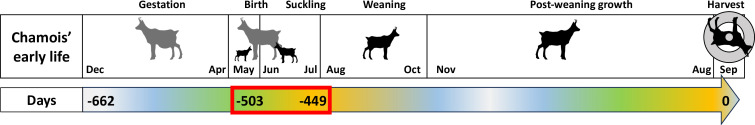
Timeline of the life stages of a yearling Alpine chamois from gestation to harvest. The timeline also features the time window reference days that have been used in the *climwin* analyses. Day −662 corresponds to 1 December of the year before the birth. The critical window identified by *climwin* spans from −503 to −449 between 9 May and 2 July of the birth year. Day 0 is 24 September of the harvest year, which corresponds to the end date of the 10-day hunting period.

We investigated the body mass of yearlings in relation to mean ambient temperature using the package *climwin* [35] in R v. 4.2.1 [36]. This package allows the detection of the precise time window when a biological variable is most strongly affected by an environmental variable by comparing the support of the data for competing hypotheses and formalizing them into regression models [37]. Competing models are based on a baseline model (not including weather effects) and ranked using the ΔAICc (difference in terms of the Akaike information criterion values calculated for a small sample size between the baseline model and the model of interest). The baseline model should include non-climatic variables that have been shown to explain variation in the response variable [37]. Therefore, ours was a multiple linear regression model with a body mass of yearling chamois as the response variable and elevation (continuous) and sex (two-level factor: male versus female) as the explanatory variables. The function *slidingwin* creates a set of competing models testing windows of different lengths for the weather variable of interest. We present results relative to the mean daily ambient temperature, but similar models using the minimum and maximum daily ambient temperatures provide similar time windows and comparable results (electronic supplementary material, S3). Nonlinear effects of temperature on body mass were investigated by testing for linear and quadratic trends. As parameters in *slidingwin*, we set an absolute time window with 24 September (the last date of harvest) as the reference day (figure 1). We looked for windows of all possible lengths, and start and end dates, between the reference day and 662 days prior (1 December of 2 years before) to include the critical periods in a young chamois’ life: gestation, suckling, first winter and yearling. Detailed information on our analyses using *climwin* is provided in electronic supplementary material, S2.

Finally, it has been reported that the climate–phenotype relationship can be potentially spurious when both variables also change across years [38]. Stronger statistical evidence for the climate–phenology relationship can be gathered by presenting a detrended relationship [38]. Therefore, we removed the temporal trends (i.e. detrending) in our climatic and phenotypic data by extracting the residuals of linear regressions between mass and year and between temperature (during the time windows previously identified using *climwin* that most strongly affect body mass) and year. We then ran a linear model with the residuals of body mass in relation to the residuals of temperature to establish that the decrease in body mass in relation to an increase in temperature was not confounded by changes in both variables across years.

## 3. Results

**Figure 2.**
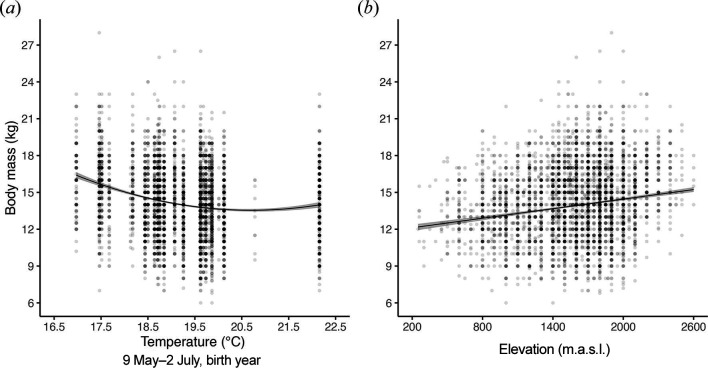
Relationship between body mass of harvested yearling Alpine chamois, (*a*) the annual average temperature between 9 May and 2 July of the birth year (suckling period) and (*b*) elevation (m.a.s.l.). Each dot is one observation (darker dots represent a higher number of observations); fitted lines in (*a*) and (*b*) show 95% confidence intervals (shaded areas).

The final model included an effect of the sex of the individual, elevation and a linear and a quadratic effect of mean daily temperature averaged between days 503 and 449 from the reference day (24 September; figure 1, table 1, electronic supplementary material, S2). This climate window is equivalent to the period from 9 May until 2 July of the birth year. It is interesting to note that the body mass of yearling chamois decreases in a nonlinear fashion as the mean annual ambient temperature increases in this critical climate window, with a substantial decrease as temperatures increase up to 19.5°C followed by a plateau at higher temperatures (table 1, figure 2*a*). Yearling chamois mass was positively related to elevation (table 1, figure 2*b*), and males were heavier than females (mean ± s.e. mass in kilograms of males: 14.2 ± 0.05; females: 13.6 ± 0.06; table 1).

**Table 1.** Results of the linear model showing the linear and quadratic effects of mean daily temperature (°C) averaged between 9 May and 2 July of the birth year (window: 503*–*449), harvest elevation (m.a.s.l.) and sex (males versus females) on body mass (kg) of harvested yearling Alpine chamois. Number of observations: 5635 in 27 years.

**predictors**	**estimate**	**s.e.**	***t***	***p*-value**
intercept	11.867	0.153	77.78	<0.001
T (window: 503–449)	−31.948	2.581	−12.38	<0.001
T (window: 503–449)^2^	14.294	3.262	4.38	<0.001
harvest elevation	0.001	0.000	14.31	<0.001
sex (M)	0.496	0.069	7.19	<0.001

**Figure 3.**
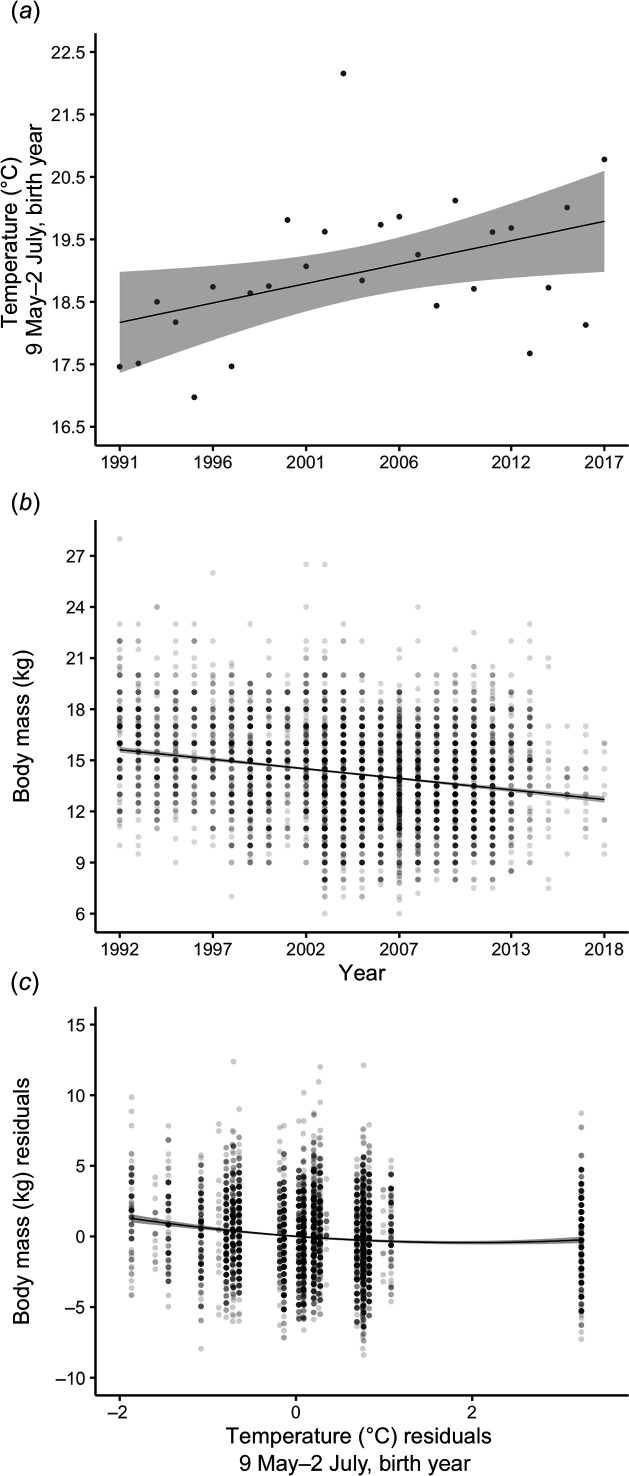
Annual trend of (*a*) mean daily temperature averaged between 9 May and 2 July of the birth year (between 1991 and 2017), (*b*) body mass of harvested yearling Alpine chamois between 1992 and 2018 and (*c*) year-detrended relationship between body mass and temperature. Detrended values in (*c*) are residuals from linear models in (*a*) and (*b*). Each dot is one observation (darker dots represent a higher number of observations in (*b*) and (*c*)); fitted lines show 95% confidence intervals (shaded areas).

Over the course of the study, the mean temperature between 9 May and 2 July increased by 0.6°C per decade (±0.2°C, *t* = 2.9, *p* = 0.007; figure 3*a*), leading to a 1.7°C increase in 27 years. During the same time period, the mean body mass of yearling chamois decreased by 1.12 kg per decade (±0.06 kg, *t* = −17.81, *p* < 0.001; figure 3*b*), leading to an overall decrease of 2.92 kg during the study period. The analysis of the relationship between body mass and mean temperature between 9 May and 2 July after detrending the data for changes across years showed a significant nonlinear negative association between both variables (both *p* < 0.001; figure 3*c*, electronic supplementary material S2), thus providing strong statistical support for an effect of temperature on body mass.

## 4. Discussion

We found that the mass of yearling chamois is influenced by a temperature window between 2 May and 21 July during the spring and summer of their first year of life, which corresponds to the end of gestation and the start of lactation. Further, we showed that the significant decrease in body mass of yearling chamois over recent decades [19] was significantly correlated with the increase in temperature between 2 May and 21 July. Our results revealed a decrease in body mass of almost 3 kg and an increase in average ambient temperature of *ca* 1.7°C between late spring and early summer during this 27-year study (1992*–*2018). The analysis of detrended data confirmed that this climate–phenotype relationship was not spurious and was simply explained by the changes in both variables across years. Our study adds to the previous research on the decrease in body mass in adults (data from this population and from Italy [19]) and yearlings (data from Italy [22] and Austria [23]). A recent study, however, did not find any change in body mass or size in chamois and three other ungulate species in the eastern Swiss Alps between 1991 and 2013 [24]. Several possible explanations are mentioned by the authors, including less extreme local changes in temperatures and forage quality in their population compared with other regions of the Alps.

Our results support the hypothesis that spring–summer temperatures are more important than winter temperatures with respect to phenotypic changes in seasonal environments [14,39]. Our study also identifies a specific critical window for chamois growth and development. Interestingly, we found a quadratic relationship with temperature in the critical time window that seems to indicate the presence of a temperature threshold for the growth of young chamois, with body mass being larger at increasingly low temperatures (note that the quadratic model is heuristic and does not imply that the relationship is parabolic over the whole range of temperatures). Future studies should further investigate this threshold in other populations and related taxa to better understand potential nonlinear relationships between climate and morphology.

Climate change can affect chamois growth in several ways. First, chamois births no longer coincide with the highest peak of vegetation growth due to rising temperatures altering the phenology of the plants they feed on [40]. The mismatch is linked to the fact that the annual birth peaks of herbivores are mainly influenced by day length, not by resource availability [41]. The lack of resources for mothers during the lactation period might influence how much energy can be allocated to nursing, with cascading effects on offspring growth. Second, although climate warming can increase plant productivity, Alpine plants are sensitive to heat stress and droughts, and climate warming is expected to decrease forage quality and palatability in Alpine grassland systems [8,9] for nursing mothers and their offspring. Third, ambient temperatures can also strongly influence the nutrient intake of yearling chamois during growth by altering the feeding activity [22] in young and adults. During warm days, chamois have been shown to reduce heat-generating activities (including foraging), probably in an attempt to avoid thermal stress [7]. Fourth, climate conditions may affect the body reserves of mothers, which in turn can affect offspring growth during gestation [42] and suckling. Ambient temperatures can also directly affect the quantity and quality of milk production [43], with increasing temperatures leading to lower milk yields in domesticated ungulates [44,45]. Climate change can also affect milk composition, with a significant decline in milk protein and fat content in response to warmer temperatures [46,47]. Finally, changes in body mass can also be driven by density-dependent effects if, for instance, chamois populations have increased in size during the study period, leading to more intense competition for access to grazing sites. This scenario is, however, unlikely as the populations of chamois have decreased in size in our study area [48] and in other parts of the Alps [40].

Our results support previous studies showing the importance of climatic conditions for ungulate growth at high elevations and latitudes [17,19,23]. Although ungulates can modify their behaviours by eating at night [49] or shifting their range to higher elevations [7] to cope with warming spring and summer temperatures, these changes in behaviour cannot cope with the negative effects of climatic conditions on forage quality and palatability [8,9], which persist throughout the day and can be observed at a wide range of elevation. Hence, studies on the effects of climate warming on the quantity and quality of forage during the time window most sensitive for ungulate growths (e.g. between 9 May and 2 July for chamois in our study population) could provide insightful information on the role of nutrition as an underlying mechanism linking climate warming to changes in body size [50]. Because body mass growth in the first few years of life can strongly impact an individual survival and future reproductive performance [12], with important consequences for population dynamics (and possibly explaining the decline in population size observed in this species [40,48]), more research is needed on the effects of climate change on body mass in the first few years of life rather than in adulthood. Consequently, it remains to be understood whether, for example, the decrease in body mass in response to the increase in summer temperatures may be detrimental to surviving the harsh variation in winter weather still present at high elevations. Research is also needed into the long-term fitness consequences of the changes in early growth on the whole life history of Alpine ungulates, from age at first reproduction to the onset of senescence and death, in order to adequately model the demographic consequences of climate change on these species.

## Data Availability

All data and code used for statistical analysis and plots are provided via the Open Science Framework and were shared with editor and reviewers at first submission [51]. Supplementary material is available online [52].
